# Trajectories of hospitalizations after age-based statutory retirement

**DOI:** 10.1007/s10433-023-00786-7

**Published:** 2023-10-28

**Authors:** Olli Pietiläinen, Jaakko Harkko, Pekka Jousilahti, Anne Kouvonen, Ossi Rahkonen, Eero Lahelma, Tea Lallukka

**Affiliations:** 1 Department of Public Health, Faculty of Medicine, University of Helsinki, Tukholmankatu 8 B, P.O. Box 20, 00014 Helsinki, Finland; 2 Faculty of Social Sciences, University of Helsinki, Helsinki, Finland; 3 Department of Public Health and Welfare, Finnish Institute for Health and Welfare, Helsinki, Finland; 4 Centre for Public Health, Queen’s University Belfast, Belfast, Northern Ireland

**Keywords:** Health outcomes, Gender, Longitudinal methods, Medical sociology

## Abstract

Retirement years are ideally spent in good health. We aimed to produce new information using person-oriented methods by identifying groups of statutory retirees who did or did not achieve this objective and the factors that distinguish these groups from each other. Our particular focus was on the years directly after the transition into retirement, and the pre-retirement factors that explained the development of health, using a more severe health-related outcome—hospitalization. We studied the retirement, hospitalizations, education, and work characteristics of former employees of the City of Helsinki, Finland (*N* = 6569), from complete registers. We used group-based trajectory models and identified groups of constant low, constant high, decreasing, and temporarily occurring hospitalizations, and one group of increasing hospitalizations among women and two groups of earlier and later increasing hospitalizations among men. Multinomial regression models showed that among women, belonging to groups with less favourable health was associated with secondary education, older age at retirement, and reduced working hours. Education and work characteristics before retirement both contribute to the development of health, as indicated by hospitalizations directly after retirement. Our findings show that socioeconomic inequalities in health are persistent and should also be addressed after transition into retirement.

**Supplementary Information:**

The online version contains supplementary material available at 10.1007/s10433-023-00786-7.

## Introduction

As populations are ageing and a significant portion of life is spent in retirement, ensuring that retirement years are spent in as good health as possible is essential, evidence on the distinct developmental patterns of health after retirement is lacking but can be obtained by identifying latent groups of individuals who maintain good health or who suffer from poor or declining health after retirement, and determining which pre-retirement factors contribute to the distinct developmental patterns in health after statutory retirement.

### The heterogeneous effects of transition to retirement on health

Transition to retirement may affect health through multiple mechanisms. Pre-retirement factors such as wealth, living environment, and life activities are unequally distributed among employees and continue so post-retirement (Hyde et al. 2004). Transition into retirement is associated with reduced income and possibly a decrease in social capital and physical activity associated with work, but on the other hand adverse work exposures are removed, and sleep duration and leisure-time physical activity often increase (Eibich 2015). Retirement may be beneficial for some domains of health but detrimental for others (Szabó et al. 2019; Xue et al. 2020). Retirement-related circumstances, such as pre-retirement work environment, lifestyle, and health, are heterogeneous, depending on the individual situation (Eibich 2015). It is unlikely there is one effect of retirement on health to be found, but there are various effects across different contexts and subpopulations which depend on personal characteristics such as education and work (Kuhn 2018; Van Ours 2023). Therefore, it is unlikely that uniform policy conclusions applicable to all prospective or current retirees can be drawn. This heterogeneity in the retirement-related mechanisms and effects of retirement on health calls for an individual-oriented approach to identify latent groups of retirees with differing health trajectories and those in need of interventions to promote or maintain good health.

Previous studies have reported conflicting findings regarding the effect of retirement on health (Van Der Heide et al. 2013). Some studies have found retirement to be beneficial for health (Jokela et al. 2010; Coe and Zamarro 2011; Eibich 2015), while others have found it to be detrimental (Dave et al. 2008; Behncke 2012; Stenholm et al. 2014). One earlier study showed that health declined before retirement, during the time just before the transition, but that this decline levelled off after retirement (Westerlund et al. 2009). The decline in health was faster among those in lower socioeconomic positions. In contrast, in our earlier study, the health of statutory retirees declined after the retirement transition, while that of disability retirees improved (Lahelma et al. 2019). A recent study has shown that self-reported life satisfaction improves during the transition into retirement, particularly among women and individuals living without a spouse (Prakash et al. 2022).

The inconsistent evidence may partly be explained by the fact that most of the above-cited previous studies have not distinguished the different routes of retirement. To be granted a disability retirement, a person needs to have an illness. Thus it is understandable and even expected that the effects on the health of disability retirees differ from those on statutory retirement. Thus, it is important to focus on statutory retirement if we are to increase our understanding of the development of health specifically after statutory retirement, and not in conjunction with other retirement routes. When the focus is on age-based statutory retirement, the selection out of the work due to poor health, i.e. the healthy worker effect (Li and Sung 1999), is less of an issue than in studies that have merged these different routes. However, findings of previous studies are not directly comparable, as most countries have different retirement arrangements, and different eligibility criteria for age-based statutory retirement. Although the heterogeneity of earlier results on the effects of retirement on health may also partly be explained by different research methodologies (Nishimura et al. 2018), the aforementioned heterogeneity across contexts and sub-populations is likely to explain much of the heterogeneous results.

### Previous findings on distinct groups of trajectories of health after retirement

To our knowledge, no previous study has examined the distinct trajectory groups of hospitalizations, which could be seen as a more severe health outcome, directly after statutory retirement. However, some studies have identified trajectories of other domains of physical health after and around retirement. Haapanen et al. (2022) examined trajectories of physical functioning among retired business executives and managers and identified five distinct groups: intact, high stable, high and declining, intermediate and declining, and consistently low. Those on the more favourable trajectories were younger and healthier, and more commonly on statutory retirement and less commonly on disability retirement than those with worse physical functioning trajectories. As only those with some of the highest occupational positions were examined, there is a need to cover the wider spectrum of occupational groups. Moreover, register-based hospitalizations and retirement as well as related diagnoses provide more accurate information on health status.

Some other studies have used self-reported measures of health. Stenholm et al. (2020) examined trajectories of self-rated health before and after statutory retirement and identified four trajectories: sustained good health, good to suboptimal health, suboptimal to good health, and sustained suboptimal health. Sustained suboptimal health was associated with low occupational class, whereas declining health was associated with low occupational class, high job strain, and low job satisfaction. Szabó et al. (2019) found three distinct trajectories in physical health functioning during retirement transition: those with stable good health until retirement and slow decline after retirement, those with poor physical health that declined until retirement and improves after retirement, and a group in the middle with initially declining physical health and slower decline after retirement. The health of those with the worst health improved after retirement but declined in the other groups. Those maintaining good health were more likely to be in a relationship and less likely to be smokers or have chronic conditions than those whose health declined or was initially poor. Groups with better health also had better economic well-being.

To sum up, few studies have examined the development of health *directly* after retirement using a person-oriented approach and more severe health outcomes such as register-based hospitalizations. Previous studies have largely focused on self-reported measures such as functioning, merged different retirement routes (i.e. disability and statutory retirement) and concentrated on specific occupations. Examining the development of health directly after retirement is important, as the health of those who have just retired and are typically in their sixties is not comparable to health of those in their seventies, eighties, or nineties. Thus, we need more information specifically about statutory retirees directly after their retirement. Moreover, we need to include objective health outcomes such as those derived from administrative records, to describe more severe health outcomes that have more significant consequences for both the individual and society. Finally, after identifying distinct trajectory groups, we can pinpoint the potentially modifiable factors or other risk factors that are associated with trajectory memberships and that could be potential targets for future interventions.

### The Finnish context

This study investigated retired municipal employees in Finland, which is a Nordic welfare state. Finland has the third oldest population in the world after Japan and Italy; 22% of the Finnish population are over the age of 65 (Population Reference Bureau PRB 2023).

In Finland, practically all employees accrue earnings-related pension. The pensionable age for the national pension is 65 for those born in 1962 or earlier (The Nordic Council and the Nordic Council of Ministers 2023). Flexible retirement is possible within the earnings-related pension scheme. Following the pension reform in 2017, the statutory retirement age rose gradually from 63 to 65. In the public sector, it is also possible for ‘old workers’ to retire at an individual or occupational retirement age according to earlier agreement.

In 2021, the employment rate of 60- to 64-year-olds in Finland was 57% (Finnish Centre for Pensions 2022). The primary outcome of this study was hospitalizations. In Finland, public healthcare, including hospital treatment, is primarily funded by taxation and subsidized, but not fully free at the point of delivery. Patient fees are fixed; they do not vary according to the actual cost of the service or the treatment. It varies somewhat between health and social care regions, but the maximum daily hospital charge in 2022–2023 was 49.60 EUR per day. However, there is a payment ceiling for public healthcare client fees (692 EUR per year in 2023). When the payment ceiling has been reached, services that count towards it are free until the end of that calendar year. However, the patient may still be charged for short-term institutional care or institutional services.

### The aims of this study

The first aim of this study was to identify distinct groups of statutory retirees who have similar health trajectories after retirement, using hospitalizations as an indicator of ill-health. The second aim was to examine the distinguishing factors of these trajectories in terms of sociodemographic, socioeconomic, and work-related explanatory factors. An additional aim was to identify the key diagnostic hospitalization groups to be able to examine whether the distinct hospitalization trajectories represent distinct diagnostic groups, providing more direct evidence for possible targeted interventions.

## Methods

### Data

The study population consisted of former employees of the City of Helsinki, Finland, who had transitioned into statutory retirement between the beginning of 2000 and the end of 2013 and had been employed for at least 5 years before retirement. We defined transition to retirement based on the date of first full-time age-based statutory retirement. It is possible that some participants re-entered the labour market after retirement or continued at work alongside retirement, but such arrangements are likely rare, and the participants were considered retired after their first retirement. The individuals were followed using registers for 5 years, starting on the first day of their retirement. In Finland, the lowest possible age for statutory retirement is typically based on birth year. All the data were register based. The City of Helsinki Personnel register contained 133,879 different employees for the years that we studied. These data could be merged with other administrative data, such as hospitalization records, using the unique identification numbers assigned to each citizen (Gissler and Haukka 2004). The final study population consisted of 6569 participants, 4796 women and 1773 men, who had taken statutory retirement from the City of Helsinki in 2000–2013 and had worked for at least 5 years before retirement. The participants were aged 55–70 on the date of their retirement. The range of retirement ages is wider than the general Finnish age of statutory retirement of 63–68 years, but our study population includes occupations such as firefighters who used to have a lower retirement age. As the earlier and later than typical age retirement dates in our data are actual age-based retirement dates, and therefore part of the cohort based on the inclusion criteria, we chose to retain them in our data. Because of their small numbers in the data, their inclusion does not distort the results.

### Outcome

We used hospitalizations for all diagnoses as the primary outcome of the study. Data on all hospital admissions were obtained from the national care register maintained by the Finnish Institute for Health and Welfare. The follow-up period was divided into 6-month intervals, and hospitalization was measured as dichotomous in each 6-month period.

For our additional analyses, the hospitalization diagnoses were available as ICD-10 codes (International Classification of Diseases 10th Revision, https://icd.who.int/browse10/2019/en). As many of the diagnostic groups in our cohort had only a small number of hospitalizations, only those that appeared within the ten most common diagnostic groups across all the identified trajectory groups were included in the analyses (please see Supplementary Figures 1–2). Thus, the diagnostic groups in the initial analysis were musculoskeletal diseases, neoplasms, diseases of the respiratory system, diseases of the circulatory system, diseases of the digestive system, diseases of the skin and subcutaneous tissue, diseases of the genitourinary system, diseases of the nervous system, diseases of the eye and adnexa, diseases of the ear and mastoid process, other abnormal symptoms, injury or poisoning, and other external causes. ICD-10 Z-codes are heterogeneous, typically indicating a need for treatment: not an identified illness, but a condition that may affect overall health (https://www.icd10data.com/ICD10CM/Codes/Z00-Z99). For clarity, seven key groups are displayed in the figures.

### Explanatory factors

Education, pre-retirement occupational class, weekly working hours, type of work contract, and age at retirement were used as explanatory factors for trajectory group membership, given their widely known and established role as social determinants of health (Lynch and Kaplan 2000; Lynch 2001; Siegrist 2004; Shaw et al. 2007). Pre-retirement occupational class was based on the job title obtained from the employer’s register. The classes were managers and professionals, semi-professionals, routine non-manual workers, and manual workers, based on the required qualifications. Pre-retirement weekly working hours were obtained from the employer’s register and were classified as 0–9, 10–30, or over 30 h per week. The type of work contract before retirement was likewise obtained from the employer’s register and classified as permanent or temporary employment. Education was obtained from the register of Statistics Finland and was classified as basic education at most, secondary school, higher vocational degree, or master’s degree or higher. Age at retirement was based on the date of retirement obtained from the Finnish Centre for Pensions register. The different data sources were linked using the national personal identification numbers of the individuals.

### Statistical analyses

#### Group-based trajectory models

Group-based trajectory models (Nagin 2005) were used to identify groups with similar trajectories of hospitalizations after retirement. First the optimal number of groups was assessed by calculating the models with two to seven groups into cubic polynomial forms. As some group membership probabilities fell below the recommended 5% (Andruff Carraro et al. 2009) in the seven group models, the next group selection process was confined to two to six models. The number and polynomial form of groups was selected according to the criteria of Nagin (2005): models with a Bayesian information criterion (BIC) closer to zero were preferred, but the chosen model had to fulfil the criteria of satisfactory classification. We used three criteria: (1) the average posterior probability of assignment had to be at least 0.7 for all the groups, (2) the odds of correct classification had to be over 5 for all the groups, and (3) the estimated group probabilities had to be close to the proportions of the sample assigned to the groups. We selected the models that fulfilled these criteria and had a sensible interpretation (see Supplementary Tables 1–2 for details).

To take into account possible selective mortality in the groups that could lead to the identification of biased trajectories and uncertain group assignments, we added a logistic model of dropout probability to the trajectory model, with the probability of dropout depending on the previous measurement (Haviland et al. 2011). Only those who died during follow-up were included in the dropout model, and missing data for other individuals were treated as missing at random. The group-based trajectory models were calculated using SAS 9.4 software’s Proc Traj package (Jones et al. 2001).

#### Multinomial regression analysis

Multinomial logistic regression models were fitted with the nnet package to enable us to examine the association between the explanatory factors and the assigned trajectory group membership (R Core Team 2021). We used a single model, and all the predictors were included simultaneously. Our supplementary analyses tested the associations of the predictors individually without adjusting the predictors with each other. Women and men were analysed separately.

Finally, our other supplementary analyses focused on different diagnoses in the selected trajectory groups for women and men, which displayed seven key diagnostic groups. More specifically, the figures show the probability of hospitalization in each diagnostic group by trajectory group, separately for women and men.

## Results

### Characteristics of the study population

Table 1 presents the characteristics of the study population. The prevalence of hospitalization was approximately equal among women and men in all the occupational classes and at all educational levels. Hospitalization was more common among those who had retired after the age of 60 than among those who had retired at a younger age. Among women, those who had worked 10–30 h per week before retirement had a slightly higher prevalence of hospitalization than those who had worked 0–9 h. Among women, those who had a permanent job contract before retirement had a slightly higher prevalence of hospitalization than those with no permanent job contract, whereas among men the difference between these groups was small.

**Table 1:** Characteristics of study population, *N* = 6569

	Women			Men		
	*N*	%	% hospitalized	*N*	%	% hospitalized
Socioeconomic position						
Managers and professionals	1218	25	71	656	37	74
Semi-professionals	889	19	73	358	20	71
Routine non-manuals	1963	41	72	132	7	73
Manual workers	726	15	68	627	35	72
Education						
Basic education at most	1341	28	68	509	29	72
Secondary school	1506	31	72	464	26	72
Higher vocational degree	1293	27	72	432	24	74
Master's degree or higher	656	14	71	368	21	72
Weekly working hours						
Over 30	3144	66	70	1173	66	72
10–30	1256	26	75	486	27	74
0–9	396	8	69	114	6	73
Job contract						
Fixed-term	262	5	65	76	4	70
Permanent	4534	95	71	1697	96	73

### Trajectories of hospitalization

**Fig. 1:**
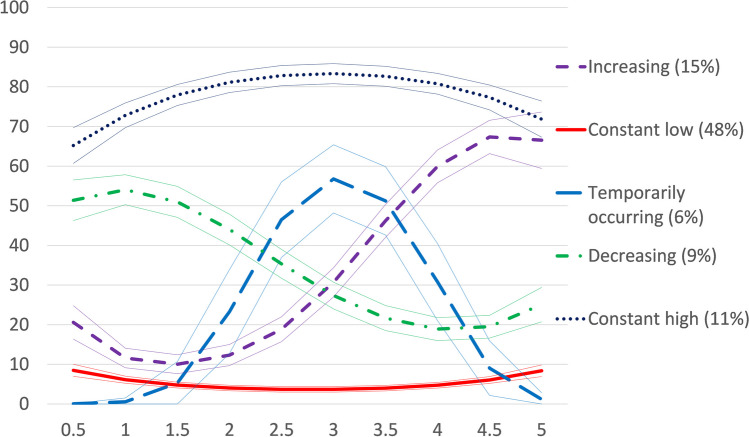
Trajectories of hospitalizations after statutory retirement, women (*y*-axis: probability of hospitalization at each time point, %; *x*-axis: ten 6-month intervals representing a 5-year follow-up)

Among women, we identified five groups with similar hospitalization trajectories after retirement (Fig. 1): (1) *increasing* (15%), (2) *constant low* (48%), (3) *temporarily occurring* (6%), (4) *decreasing* (9%), and (5) *constant high* (11%) hospitalizations. In the *constant low* group, the probability of hospitalization remained low throughout the follow-up. Among the *constant high* group, the probability of hospitalization was high already at the beginning of retirement and remained high throughout the follow-up. In the *decreasing* group, the probability of hospitalization was relatively high at the beginning of retirement but decreased towards the end of the follow-up. In the *increasing* group, the probability of hospitalization was low at the beginning of retirement but increased towards the end of the follow-up. In the *temporarily occurring* group, the probability of hospitalization was low at the beginning of retirement, began to increase around 1.5 years after retirement, reached its peak around 3 years after retirement, and decreased to almost zero towards the end of the follow-up. The relative entropy score (van de Schoot et al. 2017) was 0.72 for the model chosen for women. This did not meet the recommended cut-off of 0.8 (Nylund-Gibson and Choi 2018), but the model was deemed adequate according to average posterior probability, odds of correct classification, and because the estimated group probabilities were close to the proportions of the sample assigned to the groups.

**Fig. 2:**
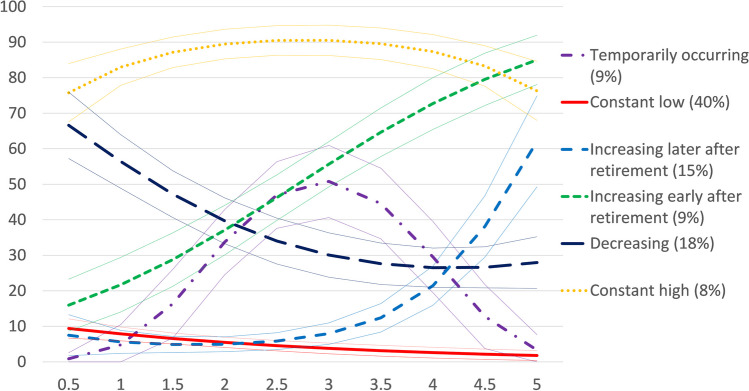
Trajectories of hospitalizations after statutory retirement, men (*y*-axis: probability of hospitalization at each time point, %; *x*-axis: ten 6-month intervals representing a 5-year follow-up)

Among men we identified six groups with similar hospitalization trajectories after retirement (Fig. 2): (1) *temporarily occurring* (9%), (2) *constant low* (40%), (3) *increasing later after retirement* (15%), (4) *increasing early after retirement* (9%), (5) *decreasing* (18%), and (6) *constant high* (8%) hospitalizations. The *constant low, constant high,* and *temporarily occurring* groups were similar to those observed among women. Whereas among women we identified only one increasing group, among men we identified two such groups, one in which the probability of hospitalization began to increase immediately after retirement, and one in which the probability of hospitalization began to increase around 3 years after retirement. The relative entropy score was 0.68 for the model chosen for men. As among women, this did not meet the recommended cut-off of 0.8 (Nylund-Gibson and Choi 2018). However, the model was deemed adequate according to average posterior probability, odds of correct classification, and because the estimated group probabilities were close to the proportions of the sample assigned to the groups.

### Factors associated with trajectory group membership

Table 2 shows how the *constant low* group was used as the reference group for examining the factors associated with the trajectory group membership among women. Having a secondary school education was associated with higher odds of belonging to the *increasing* group (OR 1.27, 95% CI 1.02–1.59), than having at most basic education. Working 10–30 h per week before retirement was associated with higher odds of belonging to the *increasing* group (OR 1.39, 95% CI 1.15–1.69) than working more than 30 h. Having a secondary school education was also associated with higher odds of belonging to the *constant high* group (OR 1.47, 95% CI 1.14–1.89). Being 60 or over at the time of the statutory retirement was associated with higher odds of belonging to the *constant high* group (OR 2.41, 95% CI 1.37–4.24).

**Table 2:** Factors associated with trajectory group membership, women (constant low as the ref.)

	Trajectory group			
	Increasing	Temporarily occurring	Decreasing	Constant high
	OR (95%)	OR (95%)	OR (95%)	OR (95%)
Socioeconomic position (ref = Professional)				
Semi-professional	1.04 (0.75, 1.43)	0.89 (0.51, 1.56)	1.16 (0.86, 1.56)	0.89 (0.62, 1.28)
Routine non-manual	1.02 (0.72, 1.45)	0.86 (0.46, 1.59)	1.07 (0.77, 1.48)	0.87 (0.59, 1.28)
Manual worker	0.83 (0.55, 1.25)	1.07 (0.54, 2.13)	1.12 (0.77, 1.62)	0.68 (0.43, 1.08)
Education (ref = Basic education at most)				
Secondary school	1.27 (1.02, 1.59)	1.11 (0.76, 1.63)	1.19 (0.97, 1.46)	1.47 (1.14, 1.89)
Higher vocational degree	1.10 (0.81, 1.50)	1 (0.58, 1.70)	1.16 (0.88, 1.54)	1.1 (0.77, 1.56)
Master's degree or higher	1.10 (0.73, 1.65)	0.92 (0.45, 1.89)	1.18 (0.81, 1.71)	0.86 (0.54, 1.37)
Age at retirement (ref = Under 60)				
60 or over	0.97 (0.67, 1.40)	1 (0.52, 1.91)	1.34 (0.92, 1.94)	2.41 (1.37, 4.24)
Weekly working hours (ref = Over 30)				
10–30	1.39 (1.15, 1.69)	1.02 (0.73, 1.44)	1.17 (0.98, 1.4)	1.06 (0.85, 1.33)
0–9	1.07 (0.75, 1.54)	0.89 (0.47, 1.68)	1 (0.72, 1.39)	0.93 (0.62, 1.4)
Permanent work contract (ref = No)				
Yes	1.18 (0.81, 1.73)	1.61 (0.74, 3.51)	0.89 (0.64, 1.22)	1.21 (0.78, 1.87)

Multinomial logistic regression analysis (odds ratios, OR, and their 95% confidence intervals (95% CI)). Fully adjusted results

In Table 3, as with the women, we used the *constant low* group as the reference group to examine the factors associated with the trajectory group membership among men. Retiring at the age of 60 or over was associated with higher odds of belonging to the *decreasing* group (OR 2.32, 95% CI 1.39–3.88) and the *constant high* group (OR 2.78, 95% CI 1.24–6.25) than retiring at a younger age. Having completed secondary school was associated with higher odds of belonging to the *increasing early* group (OR 1.67, 95% CI 1.02–2.73) than having only basic education. Having a higher vocational degree (OR 2.19, 95% CI 1.11–4.35) or master’s degree or higher (OR 2.28, 95% CI 1.01–5.12) was associated with higher odds of belonging to the *temporarily occurring* group. Working 10–30 h per week before retirement was associated with higher odds (OR 1.41, 95% CI 1.04–1.90) of belonging to the *decreasing* group than working over 30 h per week. Having a permanent work contract before retirement was associated with higher odds of belonging to the *constant high* group (OR 8.75, 95% CI 1.17–65.44). However, this group comprised only 76 men, i.e. only 4% did not have a permanent work contract.

**Table 3:** Factors associated with trajectory group membership, men (constant low as the ref.)

	Trajectory group				
	Increasing later	Decreasing	Increasing early	Temporarily occurring	Constant high
Socioeconomic position (ref = Professional)					
Semi-professional	0.73 (0.44, 1.19)	1.14 (0.72, 1.82)	1 (0.55, 1.81)	1.06 (0.55, 2.03)	0.92 (0.5, 1.7)
Routine non-manual	0.83 (0.42, 1.66)	1.76 (0.97, 3.21)	0.58 (0.22, 1.56)	0.88 (0.32, 2.41)	1.71 (0.78, 3.72)
Manual worker	1.01 (0.59, 1.73)	1.29 (0.77, 2.17)	1.17 (0.6, 2.31)	2.02 (0.97, 4.22)	0.97 (0.48, 1.96)
Education (ref = Basic education at most)					
Secondary school	1.04 (0.71, 1.53)	1.01 (0.71, 1.44)	1.67 (1.02, 2.73)	1.36 (0.82, 2.27)	0.89 (0.53, 1.51)
Higher vocational degree	1.17 (0.71, 1.94)	1.05 (0.66, 1.67)	1.87 (0.99, 3.52)	2.19 (1.11, 4.35)	1.41 (0.76, 2.63)
Master's degree or higher	0.74 (0.4, 1.37)	1.11 (0.63, 1.93)	1.28 (0.59, 2.76)	2.28 (1.01, 5.12)	1.02 (0.48, 2.18)
Age at retirement (ref = Under 60)					
60 or over	1.53 (0.95, 2.48)	2.32 (1.39, 3.88)	1.45 (0.80, 2.64)	1.17 (0.66, 2.08)	2.78 (1.24, 6.25)
Weekly working hours (ref = Over 30)					
10–30	1.22 (0.88, 1.69)	1.41 (1.04, 1.9)	0.89 (0.58, 1.37)	1.08 (0.7, 1.65)	0.99 (0.65, 1.51)
0–9	0.9 (0.46, 1.76)	1.14 (0.62, 2.09)	0.95 (0.43, 2.08)	0.84 (0.36, 1.98)	1.12 (0.5, 2.51)
Permanent work contract (ref = No)					
Yes	2.06 (0.88, 4.81)	1.29 (0.66, 2.53)	1.42 (0.56, 3.58)	1.21 (0.48, 3.04)	8.75 (1.17, 65.44)

Multinomial logistic regression analysis (odds ratios, OR, and their 95% confidence intervals (95% CI)). Fully adjusted results

Supplementary Figure 1 presents the women’s probability of hospitalization in the seven key diagnostic groups by hospitalization trajectory. The most common diagnostic hospitalization groups across the trajectory groups were musculoskeletal diseases, neoplasms, other abnormal symptoms, diseases of the circulatory system, and diseases of the digestive system. Neoplasms were especially common in the *constant high* group.

Supplementary Figure 2 presents the men’s probability of hospitalization in the seven key diagnostic groups by hospitalization trajectory. The most common diagnostic hospitalization groups across the trajectory groups were other abnormal symptoms, diseases of the circulatory system, diseases of the digestive system, musculoskeletal diseases, neoplasms, and Z-codes. Musculoskeletal diseases were especially common in the *temporarily occurring* group.

## Discussion

Our study identified five distinct hospitalization trajectory groups after statutory retirement among women and six distinct groups among men. Among both women and men, we classed the groups we identified as constant low (women 48%, men 40%), constant high (women 11%, men 8%), decreasing (women 9%, men 18%), and temporarily occurring (women 6%, men 9%) hospitalizations. We also identified a group of increasing hospitalizations among women (15%), whereas among men we identified two increasing hospitalizations groups: increasing early after retirement (9%) and increasing later after retirement (15%).

Among women, belonging to the increasing group was associated with having a secondary school education and working 10–30 h per week. Being in the constant high group was associated with having a secondary school education and retiring at the age of 60 or over. Among men, those in the decreasing group had more commonly retired at the age of 60 or over and more commonly worked 10–30 h per week than those in the constant low group. Belonging to the increasing early group was associated with lower education, while belonging to the temporarily occurring group was associated with higher education. Those in the constant high group had more commonly retired at the age 60 or over, and more commonly had a permanent work contract.

Similar studies on objectively measured health after statutory retirement using register data are either rare or non-existent. One previous study aimed to identify trajectory groups of self-reported physical health among retired managers and executives (Haapanen et al. 2022). In contrast to our study, no clear improving or declining trajectory groups were identified. However, as this study also included those on disability retirement, and only specific higher occupational groups, the results are unlikely to be comparable to ours.

Two previous studies have examined trajectories of self-rated health before and after retirement. Similarly to our study, Stenholm et al. (2020) identified trajectories with constantly good or constantly poor self-rated health after retirement, as well as improving and declining trajectories. If we examine the trajectories identified by Szabó et al. (2019) after retirement, we can see two groups of declining health and one of improving health. This differs from our results, as we found no clear groups of constant good or constant poor health. However, the changes in health of these groups were relatively small, and in sum, all the participants in the three trajectories may conflate with individuals with stable health trajectories and those with more notable changes in their health. The difference between the results of Stenholm et al. (2020) and Szabó et al. (2019) and ours may be because they included pre-retirement health in their studies, which may have affected the identification of the trajectories.

Our results give support to the notion that pre-retirement factors continue affecting health in retirement, as our results suggest that some indications of belonging to the less favourable trajectories of health after retirement as indicated by hospitalizations may be linked to work-related factors before retirement. Those in the trajectories with poorer health in particular appeared to more commonly have reduced working hours before retirement.

In the current study, older age at retirement was associated with belonging to the trajectory of constant high hospitalizations. Haapanen et al. (2022) found older age at retirement to be associated with belonging to trajectories with better health, while Szabó et al. (2019) found no association between group membership and age at retirement. Two opposing effects may be behind these contradictory results. First, older age is associated with poorer health. We did not consider age, as we focused on age at retirement and the age range was relatively narrow. However, age may have played some role in, for example, some occupational groups that had an earlier statutory retirement age in an earlier retirement system. Second, those who retired later may have been more likely to have worse health and hence more likely to have hospital admissions. Additionally, those who can continue working until an older age tend to have particularly good health. This is especially true for those in lower socioeconomic positions, i.e. manual workers and routine non-manual employees. In other words, the healthy worker effect may have played a role here (Wilcosky and Wing 1987; Li and Sung 1999). As disability retirees were included in the study of Haapanen et al. (2022), it is a strength that our focus was on statutory retirees only—a more homogeneous group who presumably retire with better health. Thus, it is difficult to make direct comparisons because the retirement age of disability retirees is typically considerably lower, and because an illness is a prerequisite for disability retirement.

Having a permanent work contract prior to retirement was also associated with belonging to the constant high hospitalizations group among the men in our study. This is somewhat surprising, as we could expect those with high levels of hospitalization to have had poorer health already before retirement, making it more difficult to maintain employment. However, those with many hospitalizations and non-permanent work contracts may have been excluded from our study, as inclusion required a long work history before retirement.

In previous studies (Szabó et al. 2019; Stenholm et al. 2020), worse trajectories of health have been associated with indicators of low socioeconomic position. In our study, education was associated with trajectory membership, but constantly high or increasing levels of hospitalization were observed not among the least educated but among the second least educated. The differences between the studies may be due to differences in the study populations. Our cohort was a municipal sector cohort from the Helsinki area, and the other two cohorts may have had wider socioeconomic and work environment structures. Although the increased levels of hospitalization were not observed among the least educated but second least educated, they were nevertheless more common in the lower educated groups than the higher educated groups, which is generally in line with the previous studies. Removal of work-related exposures on retirement would suggest that health should improve among the lower socioeconomic positions, who generally had worse working conditions, but this has not been observed in our or previous studies. Worse trajectories of health among the lower socioeconomic positions are likely to reflect the general inequalities in health, and retirement is not sufficient to erase all those inequalities.

An earlier study (Westerlund et al. 2009) found that development in health was positive after retirement, except among those whose working conditions were the most favourable before retirement. In another study (Szabó et al. 2019), retirement adversely affected the health of those who were the most affluent and the healthiest. These studies focused more on the retirement transition, and the cohorts and measures were quite different to those in our study and they used self-reported data. Nevertheless, the findings of heterogeneous effects among individual studies suggest that heterogeneity of the effects may explain contradictory results also between studies.

To assess whether the different identified trajectory groups represent different diagnostic groups, we identified the most common diagnostic groups of hospitalization in each trajectory group. The most common groups of hospitalization diagnoses in our study were musculoskeletal diseases, neoplasms, other abnormal symptoms, and diseases of the circulatory and digestive systems. The trajectory groups were mainly distinguished from each other by the number of hospitalizations, but some hospitalization diagnoses also differed. Neoplasms were especially common among women in the constant high group, whereas musculoskeletal diseases were particularly common among men in the temporarily occurring group. These diseases are common among older adults and thus among retirees in general. It is understandable that neoplasms require long-term treatments, while many musculoskeletal diseases may more easily be remedied by a shorter treatment with less hospitalizations. However, we did not examine mental diagnoses, as hospital admissions due to these are very rare, particularly in this group of individuals, who continued working until their statutory retirement.

Certain limitations of our study warrant mentioning. First, as this study was based exclusively on register data, the information available in the registers was limited. Therefore, we were not able to include important risk factors or potential confounders such as health behaviours or working conditions in our analysis, although occupational socioeconomic position and education, which were included in the analyses, may act as partial proxies for these risk factors. In addition to these, demographic factors such as marital status and number of children could be of importance. Second, as our results are based on data on previously employed municipal workers with long working careers, they cannot be generalized to the general population. Future studies could aim to identify whether developmental patterns in health are similar after retirement from the private sector, for example. However, the public sector is very large in Finland, covering around a fifth of all salaried employees, and our cohort included thousands of occupational titles, representing different socioeconomic positions and both manual and non-manual work. Third, this study focused on age-based statutory retirement, excluding disability retirement which is a path out of employment for many, especially those in lower socioeconomic positions (Polvinen et al. 2013). Hospitalization trajectories after disability retirement therefore warrant further research. Fourth, the method we used for identifying the groups of hospitalizations—group-based trajectory modelling—involves a certain arbitrariness in group identification, as there is no one correct configuration of trajectories and therefore no unambiguous way of finding it. However, this is not a limitation of the method per se, but a feature of the problem domain: there were no unambiguously distinct groups in the study population, and the aim of the study was to find a model that captures the essential features of the data. To achieve this in as objective a way as possible, we followed the commonly used guidelines of model selection and model adequacy testing found in the literature. Fifth, health before retirement was not included, as it might lead to overadjustment. Prior health is linked to future health. Thus, the identified trajectories are likely to reflect prior health. We were specifically interested in how health develops directly after retirement and used a more severe health outcome, i.e. hospitalizations, which has not been investigated before. It is very likely that those with increasing hospitalizations had poorer functioning and self-rated health as well as poorer health behaviours and working conditions before retirement. As we were able to include education, this may have partly captured these health inequalities before retirement transition. In all, these questions about the effects of pre-retirement health on later health warrant a separate study and are likely more relevant for those retiring due to disability, for which a health problem before retirement is a prerequisite. Finally, it is not possible to fully rule out whether the results are due to later retirement or an age effect. This is because the group-based trajectory models (GBTM) model is typically run without covariates, and adding a time-invariant covariate such as birth year would merely test whether it correlated with trajectory membership. Thus, in our multinomial logistic regression models, we show how age and other covariates are associated with trajectory membership.

Our study also has strengths. First, we used extensive register-based data comprising the entire workforce of the City of Helsinki, the largest employer in Finland with hundreds of occupational titles across the socioeconomic spectrum. Second, our measure of ill-health, i.e. hospitalization, was based on objective data from registers. This makes it more reliable as we did not need to rely on self-reported data on symptoms only, for example. Third, selective mortality during follow-up may cause selective attrition and distort the identified trajectories if it is unaccounted for. However, we were able to mitigate this by including information on deaths acquired from registers in a dropout model. Thus, the reported outcome should reflect the development of health after retirement more reliably for this group.

## Conclusion

We produced new information identifying distinct groups of hospitalization trajectories after retirement, with different social determinants. Education and earlier work characteristics contributed to the development of hospitalizations after retirement, which highlights the persistence of socioeconomic inequalities in health after the transition into retirement. These inequalities, specific risk factors, and the prevention of the common diagnoses for hospitalizations should be considered.

### Supplementary Information

Below is the link to the electronic supplementary material.

Supplementary file1 (PDF 983 kb)

## Data Availability

The data sets generated and analysed during the current study are not publicly available because the register data are only available from the register data holders, based on application and the Act on the Secondary use of Health and Social Data. The data cannot be made publicly available due to strict data protection laws.
