# Supplementary material for: Trajectories of hospitalizations after age-based statutory retirement
Source: Eur J Ageing. 2023 Oct 28;20(1):41. doi: 10.1007/s10433-023-00786-7 (PMC10613168; doi:10.1007/s10433-023-00786-7)
Supplement: Supplementary file 1 — Supplementary file1 (PDF 983 kb) [file 10433_2023_786_MOESM1_ESM.pdf]

Supplementary materials

Trajectories of hospitalizations after age-based statutory retirement

Olli Pietiläinen, PhD, Jaakko Harkko, PhD, Pekka Jousilahti, Professor, Anne Kouvonen, Professor, Ossi Rahkonen, Professor, Eero Lahelma Professor, and Tea Lallukka, Professor

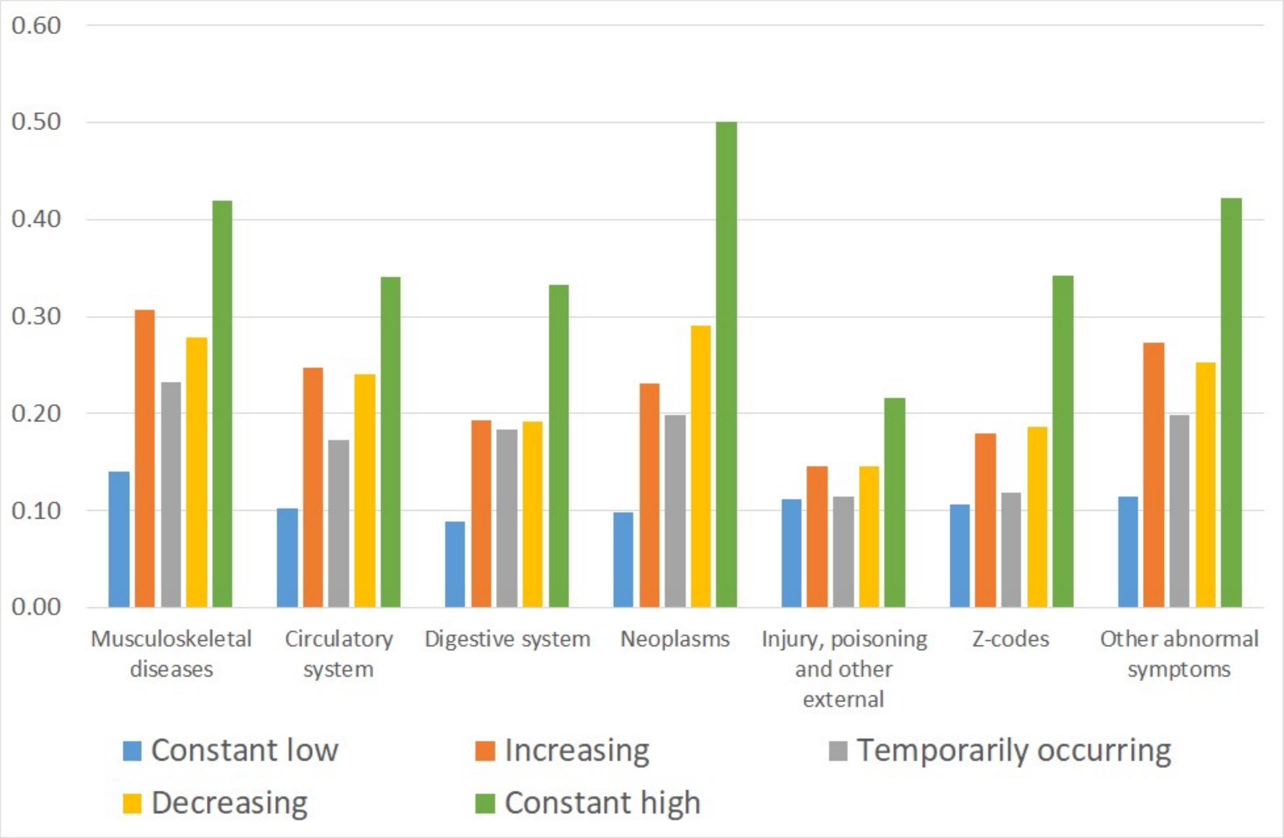

Supplementary Figure 1. Probability of hospitalization in each diagnostic group by trajectory group, women. ICD-10 Z-codes are heterogeneous, typically indicating need for treatment that is not an identified illness. The condition may, however, affect overall health (https://www.icd10data.com/ICD10CM/Codes/Z00-Z99)

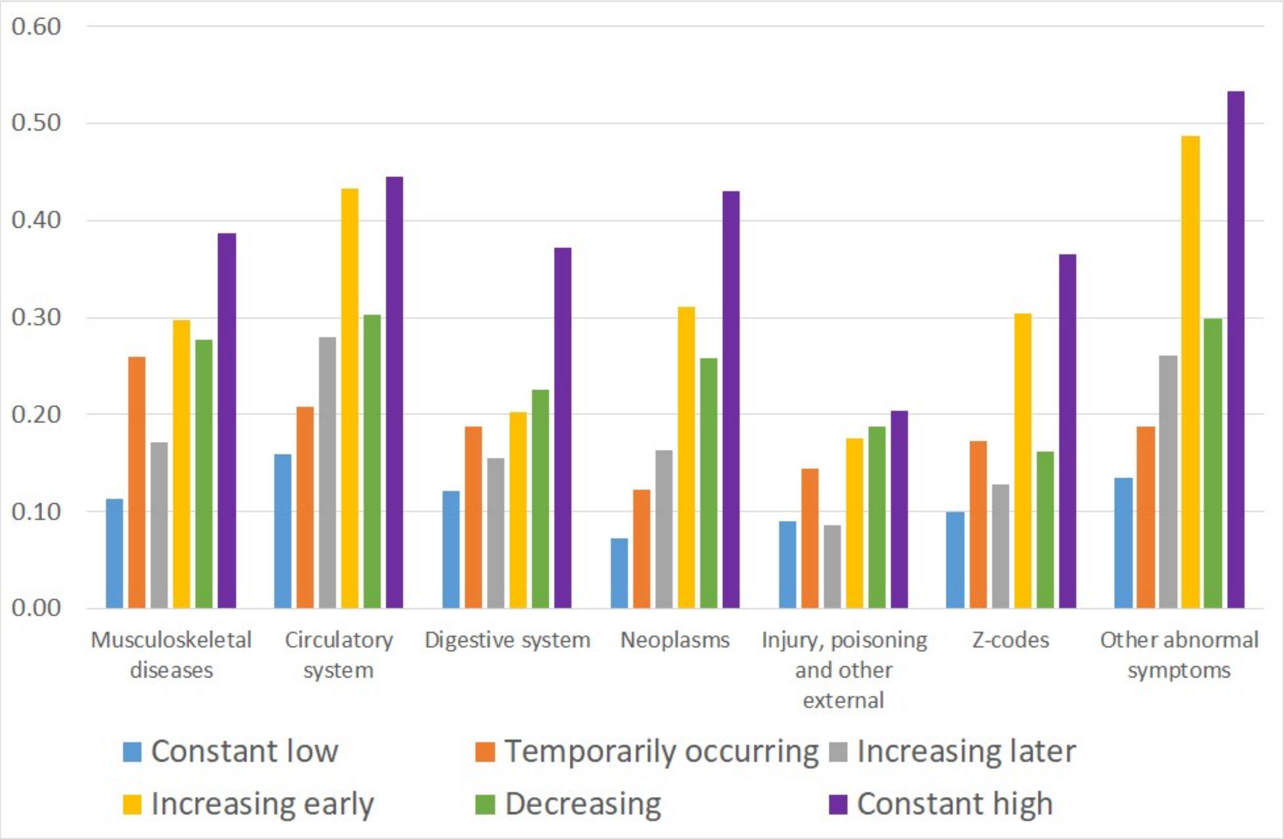

Supplementary Figure 2. Probability of hospitalization in each diagnostic group by trajectory group, men.

ICD-10 Z-codes are heterogeneous, typically indicating need for treatment that is not an identified illness. The condition may, however, affect overall health (https://www.icd10data.com/ICD10CM/Codes/Z00-Z99)

Supplementary table 1: Criteria for model selection in trajectory analysis, women. Number of groups, trajectory shapes, Bayesian information criterion, and group sizes in percentage. Selected model in bold.

Number of groups	Trajectory shapes[a]	BIC[b] (N=47546)	BIC[c] (N=4796)	Group 1 percentage	Group 2 percentage	Group 3 percentage	Group 4 percentage	Group 5 percentage	Group 6 percentage	Group 7 percentage
2	3 3	-23559	-23574	71	29					
3	3 3 3	-23168	-23191	50	39	11				
4	3 3 3 3	-22880	-22911	50	18	21	10			
**5**	**3 2 2 3 2**	**-22810**	**-22845**	**15**	**48**	**6**	**20**	**11**		
5	3 3 3 3 3	-22833	-22872	23	37	10	22	8		
6	3 2 3 3 3 3 [d]	-22760	-22806	6	46	12	8	21	7	
6	3 3 3 3 3 3 [d]	-22764	-22811	7	45	12	8	21	7	
7	3 3 3 3 3 3 3	-22750	-22805	7	19	32	10	21	8	3 [e]

[a] Model shapes: 2=quadratic, 3=cubic

[b] Bayesian information criterion (BIC) on longitudinal level

[c] Bayesian information criterion (BIC) on subject level

[d] Model not chosen due to the average posterior probability of assignment being under the recommended minimum of 70% in some groups

[e] Model not chosen due to group size being under recommended minimum of 5%

Supplementary table 2: Criteria for model selection in trajectory analysis, men. Number of groups, trajectory shapes, Bayesian information criterion, and group sizes in percentage. Selected model in bold.

Number of groups	Trajectory shapes[a]	BIC[b] (N=17430)	BIC[c] (N=1773)	Group 1 percentage	Group 2 percentage	Group 3 percentage	Group 4 percentage	Group 5 percentage	Group 6 percentage	Group 7 percentage
2	3 3	-8885	-8900	73	27					
3	3 3 3	-8759	-8782	57	35	8				
4	3 3 3 3	-8674	-8705	52	18	20	10			
5	3 3 3 3 3	-8653	-8691	17	40	15	20	8		
**6**	**2 1 2 1 2 2**	**-8633**	**-8670**	**9**	**40**	**15**	**9**	**18**	**8**	
6	3 3 3 3 3 3	-8650	-8697	26	16	21	14	16	7	
7	3 3 3 3 3 3 3	-8647	-8702	9	14	34	9	19	11	5

[a] Model shapes: 1=linear, 2=quadratic, 3=cubic

[b] Bayesian information criterion (BIC) on longitudinal level

[c] Bayesian information criterion (BIC) on subject level

Supplementary table 3. Factors associated with trajectory group membership, women (Constant low as the ref.). Multinomial logistic regression analysis (odds ratios, OR, and their 95% confidence intervals, 95% CI). Unadjusted results (each explanatory factor in separate model).

	**Trajectory group**			
	Increasing	Fluctuating low	Slow decrease	Constant high
**Socioeconomic position**				
Semi-professional	1.01 (0.79,1.30)	0.98 (0.63,1.54)	1.1 (0.88,1.39)	1.02 (0.77,1.36)
Routine non-manual	1.03 (0.84,1.27)	0.99 (0.68,1.43)	0.98 (0.81,1.19)	1.06 (0.84,1.35)
Manual worker	0.81 (0.61,1.07)	1.23 (0.79,1.91)	1.01 (0.79,1.29)	0.81 (0.59,1.12)
**Education**				
Secondary school	1.30 (1.05,1.62)	1.06 (0.73,1.53)	1.15 (0.94,1.40)	1.45 (1.13,1.85)
Batchelor's degree	1.16 (0.92,1.46)	0.96 (0.65,1.41)	1.13 (0.92,1.39)	1.23 (0.94,1.59)
Master's degree or higher	1.18 (0.89,1.55)	0.92 (0.57,1.49)	1.10 (0.86,1.41)	1.04 (0.75,1.45)
**Age at retirement**				
60 or over	0.95 (0.66,1.35)	1.01 (0.54,1.91)	1.31 (0.91,1.89)	2.12 (1.21,3.71)
**Weekly working hours**				
10 to 30	1.36 (1.13,1.64)	1.03 (0.73,1.43)	1.17 (0.99,1.40)	1.08 (0.86,1.34)
0 to 10	1.05 (0.77,1.42)	0.87 (0.50,1.51)	0.99 (0.74,1.31)	0.93 (0.65,1.33)
**Permanent work contract**				
Yes	1.13 (0.78,1.65)	1.65 (0.76,3.58)	0.88 (0.64,1.21)	1.18 (0.76,1.83)

Supplementary table 4. Factors associated with trajectory group membership, men (Constant low as the ref.). Multinomial logistic regression analysis (odds ratios, OR, and their 95% confidence intervals, 95% CI). Unadjusted results (each explanatory factor in separate model).

	**Trajectory group**				
	Increasing later	Decreasing	Increasing early	Temporary	Constant high
**Socioeconomic position**					
Semi-professional	0.89 (0.60,1.33)	1.06 (0.74,1.53)	1.06 (0.66,1.70)	0.82 (0.48,1.37)	0.97 (0.59,1.58)
Routine non-manual	0.92 (0.51,1.66)	1.5 (0.92,2.43)	0.55 (0.23,1.34)	0.58 (0.24,1.42)	1.43 (0.75,2.71)
Manual worker	1.08 (0.78,1.5)	1.02 (0.75,1.40)	0.99 (0.66,1.50)	1.11 (0.74,1.67)	0.76 (0.49,1.18)
**Education**					
Secondary school	1.02 (0.70,1.49)	1.03 (0.73,1.47)	1.58 (0.97,2.57)	1.26 (0.76,2.08)	0.88 (0.53,1.49)
Batchelor's degree	1.16 (0.79,1.69)	1.01 (0.7,1.46)	1.69 (1.03,2.78)	1.37 (0.82,2.28)	1.43 (0.89,2.32)
Master's degree or higher	0.82 (0.54,1.25)	1.02 (0.7,1.47)	1.20 (0.7,2.06)	1.37 (0.81,2.30)	1.08 (0.64,1.81)
**Age at retirement**					
60 or over	1.48 (0.93,2.34)	2.34 (1.43,3.85)	1.41 (0.8,2.50)	1.14 (0.66,1.98)	2.96 (1.35,6.51)
**Weekly working hours**					
10 to 30	1.27 (0.92,1.74)	1.49 (1.11,1.99)	0.96 (0.64,1.46)	1.12 (0.74,1.68)	1.11 (0.73,1.68)
0 to 10	0.83 (0.45,1.53)	0.98 (0.56,1.69)	0.96 (0.47,1.95)	0.84 (0.39,1.83)	0.97 (0.46,2.03)
**Permanent work contract**					
Yes	2.09 (0.93,4.70)	1.35 (0.72,2.56)	1.38 (0.58,3.31)	1.30 (0.54,3.11)	7.96 (1.09,58.27)
